# Effect of Dental Status on Changes in Mastication in Patients with Obesity following Bariatric Surgery

**DOI:** 10.1371/journal.pone.0022324

**Published:** 2011-07-20

**Authors:** Anne Espérance Godlewski, Jean Luc Veyrune, Emmanuel Nicolas, Cécile A. Ciangura, Catherine C. Chaussain, Sébastien Czernichow, Arnaud Basdevant, Martine Hennequin

**Affiliations:** 1 Clermont Université, Université d′Auvergne, EA 3847, Clermont-Ferrand, France; 2 CHU de Clermont-Ferrand, Clermont-Ferrand, France; 3 Hôpital Pitié Salpêtrière, Pôle d′endocrinologie, AP-HP, Paris, France; 4 Université Paris Descartes, EA 2496, Université Paris-Descartes, Paris, France; 5 Hôpital Bretonneau, Service d′Odontologie, AP-HP, Paris, France; 6 Hôpital Ambroise Paré, Département de Nutrition, Université de Versailles, St-Quentin, Boulogne-Billancourt, France; 7 Centre de Recherche en Epidémiologie et Santé des Populations, INSERM U1018, Villejuif, France; 8 Université Pierre et Marie Curie, INSERM U755, IFR58, Paris VI, Paris, France; Paris Institute of Technology for Life, Food and Environmental Sciences, France

## Abstract

**Background:**

Patients scheduled for bariatric surgery (BS) are encouraged to chew slowly in order to optimise the digestion process. The influence of dental status on patients' ability to comply with advice on chewing behaviour is poorly documented. This study aims to compare modifications of chewing function before and after BS in three groups of obese patients differing in dental status.

**Method and Findings:**

A cohort of 46 obese women provided three groups: FD group: fully dentate (7–10 functional dental units [FU]); PD group: partially dentate (4–6 FU) without partial dentures; DW group: partial and complete denture wearers. Chewing time (CT), number of chewing cycles (CC), and chewing frequency (CF) were measured before and after surgery during mastication of standardised samples of raw carrot, peanuts, banana, apple and jelly. The median particle-size distribution (D50) of the pre-swallowed bolus was also evaluated for peanut and carrot. Before surgery, the PD and DW groups exhibited greater mean CCs and CTs than the FD group (SNK p<0.05) and produced a bolus with higher granulometry (SNK, p<0.05) than the FD group. After surgery, CT and CC increased for all groups and for all foods, but not statistically significant for jelly. The resulting changes in bolus granulometry observed depended on both food and dental status. The granulometry of carrot bolus remained as fine or as coarse in FD and DW groups respectively as it was before surgery while it was significantly decreased in the PD group (Student's test, p<0.001).

**Conclusions:**

After bariatric surgery, all the obese patients, regardless of dental status modified their chewing kinematics. The effects of this chewing behaviour on bolus granulometry depended on dental status and type of food. Further studies are needed to understand better the impact of dental status on feeding behaviour and nutrition in patients with obesity.

## Introduction

Bariatric surgery (BS) is the only consistently-effective long-term treatment for morbid obesity [1]. Preliminary state-level data from the USA cite the number of bariatric surgical procedures at approximately 130,000 in 2005, with a forecast of 218,000 in 2010 [2]. In France, figures from 2008 indicate that the number of bariatric surgical operations (BS) carried out that year was 13,722 [3]. This widespread solution results in significant weight loss and reduces obesity-related co-morbidities, with an acceptable rate of short- and long-term side effects [4], [5]. Its efficacy is based on both the restriction of the quantities of ingested food and the malabsorption of the nutrients through the shunted gut.

International guidelines generally suggest that patient criteria for elective surgery for BS should firstly, include the control of feeding behaviour in order to eat less and more slowly and, secondly, a functional dental status providing good chewing function [6]. This set of conditions was assumed to reduce the prevalence of physiological complications of BS, such as vomiting, diarrhoea, pain or dumping syndrome. The relationships between mastication and digestion have previously been investigated in different ways [7]. Increasing mastication shortens the time needed by the stomach to comminute food particles to a diameter small enough to pass through the pylorus [8]. Mastication is also involved in maintaining good motility in the digestive tract by enhancing physiological gastric motion through the activation of parasympathetic nervous activity [9]–[14]. Moreover, adequate mastication facilitates the initial steps of digestion by stimulating saliva production and activating the cephalic controls that initiate the assimilation of foods [15], [16].

Chewing ability in persons with obesity could affect links between nutrition and feeding behaviour. Previous studies on feeding attitudes demonstrated that obese subjects eat faster than their lean peers and suggested that a lack of oral stimulations could be related to energetic metabolism [17], [18]. It has been suggested that low activity of the autonomous nervous system explains a decrease in the thermogenic response to food in individuals with obesity [19], [20]. It was also shown that the palatability of the meal had an effect on the cephalic phase of dietary thermogenesis and that this effect is significantly decreased in obese subjects compared with non-obese ones [21]. Patients with morbid obesity who have undergone bariatric surgery are thus encouraged to chew slowly in order to slow down food intake and optimize the digestion process.

Despite these considerations, BS is often proposed for obese patients whatever their chewing ability. A previous study, conducted in a group of 44 patients with morbid obesity scheduled for BS, showed that 43% had healthy dental status with at least 7 pairs of functional teeth (Functional Unit: FU), while 23% had 6 FUs, 20% had 5 FUs and 14% wore dentures [22]. In this latter study, it was shown that patients with impaired dental status produced a food bolus with a larger particle size than fully dentate patients. It was not known however whether the patients with impaired dental status were able to comply with advice about increasing chewing and, if such a modification were verified, whether an increase in chewing activity had an impact on the composition of the swallowed bolus.

The observations of both the food bolus collected just before swallowing and the kinematic strategy developed to produce this bolus appeared to be good criteria for evaluating mastication [23], [24], [25]. It has been demonstrated in healthy adult subjects that the granulometry of the bolus decreases during chewing until reaching a threshold value that corresponds to swallowing [26], [27]. This threshold is a constant of each individual and depends on food rheological properties [28], [29]. It varies among individuals according to dental status and saliva characteristics and its variability is not affected by gender [30]. In healthy fully dentate subjects, the chewing activity before swallowing differs according to type of food, with more chewing cycles (CC) and longer chewing duration (CT) for hard foods such as carrot and peanuts than for soft foods such as banana and apple, while the chewing frequency (CC/CT) of each individual remains constant [30]–[33]. Previous studies on the chewing ability of dentally impaired subjects showed that a decrease in the number of functionally paired teeth and oral rehabilitation with removable dentures were linked to a decrease in CT and CC values and to an increased D50 value [22], [30].

In obese patients, the impact of potentially increased chewing activity on the granulometry of the pre-swallowed bolus would also depend on dental status. The present study aims to address this point by comparing chewing parameters and the granulometry of the pre-swallowed bolus before and after gastric bypass surgery in patients with morbid obesity scheduled for bariatric surgery and differing in dental status. Comparisons between pre- and post-surgery data will be analysed in order to validate the following hypotheses: 1°) Fully dentate patients increase their chewing activity, while bolus granulometry, which has already reached its optimal value for triggering the swallowing reflex, does not change. Consequently we expected an increase of post-surgery CT and CC values compared with pre-surgery CT and CC values, while the post-surgery D50 values were expected to be close to the pre-surgery D50 values; 2°) For partially dentate patients, we expected increased chewing activity with the highest CC and CT values which, finally, should produce a lower bolus granulometry than before surgery, with mean post-surgery D50 values in the PD group being close to the standard values of the FD group; 3°) For the DW group, both obesity and denture wear are factors that led patients to increase their chewing activity (CC and CT) without any reducing effect on bolus granulometry. Regarding this situation, we do not have any predictive information on the adaptive abilities of patients with morbid obesity who wear full dentures. More particularly we do not know whether or not such patients are able to increase their chewing activity again or not. We also do not know the impact of a possible increase in chewing activity with dentures on bolus granulometry.

## Methods

This prospective observational study was conducted on a group of patients with morbid obesity for whom bariatric surgery was indicated in the Department of Nutrition, Salpêtrière Hospital (Paris, France). The study was approved by the local ethics committee (CPP: 2007/12) and all the subjects gave informed consent.

### Study participants

Patients with morbid obesity scheduled for bariatric surgery between September 2005 and March 2007 were proposed for inclusion. Morbid obesity has been defined as a body mass index exceeding 40 kg /m2 (or ≥35 kg/m2 with a serious factor of co- morbidity) [34]. Patients with mental, cognitive or neurological diseases, infectious oral diseases such as caries or periodontal disease, or without dentures despite having fewer than four dental functional units, were not included. All patients received instructions on diet, food consumption and advices on chewing activity in accordance of international guidelines [6].

### Study Criteria

#### Anthropometric status

Age, gender, height and weight were recorded at each session. Body Mass Index (BMI) was calculated from the formula: Weight (kg) /Height (m)^2^.

#### Dental Status

After examination by a dentist, patients were categorized according to the number of functional dental units (FU), defined as a pair of posterior antagonist natural teeth having at least one contact area during chewing and according to whether they wore a partial or complete denture. The FU number was evaluated by asking the subjects to chew 1–2 cycles on articulating paper 200 µm thick; the number of teeth on the mandibular arch that had at least one coloured mark gave the number of FUs.

Three groups were formed: i) the fully dentate group (FD group) included all patients with 7 to 10 FU; ii) the partially dentate group (PD group) included all patients with 4 to 6 FUs without partial dentures, iii) the denture wearer group (DW group) included all patients with from 0 to 3 natural FU and wearing partial or complete dentures. Patients with fewer than 3 natural teeth without dentures were excluded.

#### Chewing criteria

The kinematic parameters of mastication and the granulometry of the bolus were assessed during a food chewing test session. All the patients were scheduled for the same evaluation before and three months after bariatric surgery.

*Kinematic parameters*: Six natural foods with different rheological properties were proposed for mastication during a chewing session. Two standardized samples of jelly, banana, apple, carrot (cylinders 2 cm diameter adjusted in height to weigh 4±0.5 g) and unsalted raw peanuts (selected to weigh 4±0.5 g) were first proposed for chewing and swallowing in random order. A digital camera positioned in front of the subject (face-on) made a video recording of the face. The evaluations of each variable required an independent reading of each recording by a calibrated observer who watched the videos in random order. The method had previously been validated for healthy fully dentate patients and for denture wearers [35], [36]. The indicators recorded were chewing time (CT: the time in seconds between the moment when the food was placed in the mouth and swallowing, identified by swallowing immediately after the end of rhythmic rotary movements) and the number of chewing cycles (CC: number of chewing actions during the CT period; this included all the rotary patterns, with and without lip closure). Chewing frequency (CF) was calculated as the ratio CC/CT.

*Bolus granulometry*: After the chewing sequences recorded for video measurement, the patients were asked to chew three additional replicates of the carrots and peanuts and to expectorate each bolus when they thought they were ready to swallow it. During the chewing of each replicate, CT was monitored by an investigator and compared with the CT value previously recorded during the collection of the kinematic parameters. If there was a difference greater than ±5 s, the patient was asked to chew a new test food piece. Each chewed bolus (masticate) was collected in a container, rinsed with water on a 100-μm sieve to eliminate saliva, and dried at 80°C for 30 min. The masticate was then spread onto a transparent A4 sheet. The sheet was scanned to produce a 600-dpi image (Epson perfection 4990 photo™). The images were then processed by software to evaluate food particle size and distribution (Powdershape®, Innovative Sintering Technologies). For each masticate, the results were expressed in terms of the D50 value characterizing the theoretical sieve size that would allow 50% of the particles to pass. Thus the D50 value decreased as bolus particle distribution became finer.

### Data analysis

Statistical analysis was performed using IBM®-SPSS®19 software; statistical significance was set at *p* <0.05. Mean CT, CC, CF, and D50 values were calculated for each group of obese subjects before and after surgery. Verified check was performed on whether each variable was normally distributed. To assess the impact of dental status on the ability to chew and to evaluate whether the chewing pattern was modified by the instructions on increasing chewing activity, statistical analysis was carried out to detect any differences in mean chewing parameters between the subgroups of patients. For inter-group comparisons of mean presurgical D50, CT, CC and CF values, a General Linear Model procedure (dependant factors: D50, CT, CC and CF; fixed factors: dental status, type of food) followed by the post-hoc Student Newman-Keuls (SNK) were applied. The mean D50, CC, CT and CF values measured pre- and post-operatively were compared using Student's paired *t*-test to evaluate the impact of surgery period on chewing parameters. An ANOVA was applied to assess the impact of dental status on mean % BMI variations.

## Results

Among the 140 patients recruited over 18 months for a dental check-up, 46 women and 8 men agreed to participate in both pre- and post-surgical chewing evaluations (women: mean age 41.9±11.3 years, 95% CI [38.3, 47.4]; men: mean age 46±9.8 95% CI [37.7, 54.2]. Considering the variations of CC, CT and CF with gender [29], all data from male patients were excluded from this analysis. The patient distribution range according to dental status, age, mean BMI and proportional BMI variations before and after surgery are reported in Table 1. The mean BMI variations recorded 3 months after surgery do not differ significantly between the groups of patients on the basis of dental status (ANOVA, F = 0.5, P = 0.59). Calculations demonstrated that a difference in BMI variations according to dental status could be tested with the inclusion of 4882 subjects per group (α = 0.05; β = 0.10). The proportion of variations and the mean values of CC, CT and D50 values recorded before and after surgery in each group of patients are reported in Table 2.

**Table 1:** Distribution of patients included in the study according to dental status, age, BMI, and proportionate variation in BMI at three months post-surgery.

Group	Number of subjects	AGE (years)		Mean BMI before surgery		Mean BMI at 3 months post-surgery		Mean % of BMI variation	
		Mean	*±SD*	Mean	*±SD*	Mean	*±SD*	Mean	*±SD*
FD group (7–8 FU)	23	39.8	*11. 8*	46.3	*5.3*	37.6	*5.6*	-18.9%	*6.9*
PD group (4–6 FU)	15	44.3	*11.9*	48.6	*9.7*	39.6	*9.7*	-19.8%	*6.8*
DW group (O–3FU and Denture)	8	46.3	*11.0*	49.8	*12.1*	41.4	*8.7*	-16.4%	*3.9*
Total	46	42.2	*11.8*	47.7	*8.3*	39.9	*7.7*	-18.8%	*6.7*

**Table 2:** Means values (*±SD*) of number of chewing cycles (CC), chewing time (CT) and median particle size of the pre-swallowed bolus (Bolus granulometry, D50) recorded before and after surgery in each group of patients.

Food	Dental status groups	Chewing Time CT (s)			Chewing Cycles CC			Chewing Frequency CF (cycles/s)			Blus granulometry D50 (µm)		
		Before	After	P	Before	After	P	Before	After	P	Before	After	P
Carrot	FD group	23.6	30.6	***	36.1	47.0	***	1.6	1.6	ns	2454	2506	ns
		(*10.9*)	(*12.2*)		(*15.9*)	(*18.5*)		(*0.2*)	(*0.2*)		(*812*)	(*486*)	
	PD group	31.1	40.2	***	44.4	58.9	***	1.5	1.5	ns	4015	3086	***
		(*13.8*)	(*17.2*)		(*20.3*)	(*27.8*)		(*0.3*)	(*0.2*)		(*963*)	(*836*)	
	DW group	31.1	38.0	***	44.4	54.5	***	1.5	1.5	ns	3531	3410	ns
		(*12.7*)	(*17.3*)		(*16.6*)	(*22.1*)		(*0.2*)	(*0.2*)		(*656*)	(*330*)	
Peanuts	FD group	29.2	33.1	***	43.4	48.8	***	1.5	1.5	ns	1560	1886	**
		(*13.4*)	(*12.8*)		(*20.0*)	(*19.0*)		(*0.2*)	(*0.6*)		(*480*)	(*834*)	
	PD group	34.7	42.7	***	47.4	62.5	***	1.4	1.5	*	2556	2494	ns
		(*15.8*)	(*18.8*)		(*23.5*)	(*27.5*)		(*0.3*)	(*0.2*)		(*700*)	(*757*)	
	DW group	35.5	41.9	**	49.5	58.2	***	1.4	1.4	ns	2321	2894	ns
		(*18.1*)	(*19.9*)		(*27.6*)	(*29.9*)		(*0.2*)	(*0.3*)		(*812*)	(*897*)	
Banana	FD group	9.2	12.5	***	11.7	14.4	***	1.3	1.2	ns			
		(*3.6*)	(*5.8*)		(*4.3*)	(*6.0*)		(*0.2*)	(*0.4*)				
	PD group	9.5	12.2	***	10.1	14.3	***	1.1	1.1	ns			
		(*6.1*)	(*7.6*)		(*6.6*)	(*11.7*)		(*0.2*)	(*0.3*)				
	DW group	9.0	10.1	ns	10.3	11.2	ns	1.1	1.1	ns			
		(*4.7*)	(*2.4*)		(*5.0*)	(*3.2*)		(*0.3*)	(*0.2*)				
Jelly	FD group	36.5	41.2	ns	43.7	50.6	ns	1.2	1.5	ns			
		(*14.1*)	(*19.9*)		(*20.1*)	(*21.3*)		(*0.2*)	(*1.5*)				
	PD group	35.1	39.6	ns	37.9	43.2	ns	1.1	1.1	ns			
		(*15.9*)	(*17.5*)		(*14.2*)	(*17.4*)		(*0.2*)	(*0.2*)				
	DW group	63.7	61.2	ns	68.5	65.5	ns	1.1	1.1	ns			
		(*52.2*)	(*41.1*)		(*63.9*)	(*45.8*)		(*0.2*)	(*0.2*)				
Apple	FD group	12.1	17.8	**	16.4	27.8	***	1.4	1.5	ns			
		(*4.6*)	(*9.2*)		(*6.9*)	(*16.6*)		(*0.3*)	(*0.3*)				
	PD group	13.3	18.1	**	17.0	22.7	**	1.3	1.3	ns			
		(*8.7*)	(*10.1*)		(*9.5*)	(*11.6*)		(*0.2*)	(*0.3*)				
	DW group	13.9	16.0	ns	17.3	19.1	ns	1.3	1.3	ns			
		(*4.8*)	(*7.3*)		(*6.2*)	(*7.8*)		(*0.3*)	(*0.3*)				

FD group (n = 23): patients with at least 7 functional dental units; PD group (n = 15): patients with 4, 5 or 6 functional dental units without dentures; DW group (n = 8): patients with fewer than 4 functional dental units wearing dentures. Student's paired *t*-test was used for respective comparisons of pre- and post-surgical means (ns: not significant; *: p<0.05; **: p<0.01; ***: p<0.001).

Before surgery, the chewing activity of partially dentate patients and denture wearers was greater than that of fully dentate patients. GLM procedure showed that the mean values of CC (F = 15; p<0.001), CT (F = 7; p = 0.001) and CF (F = 15; p<0.001) varied with dental status; these variations differed by food type for CC (F = 75; p<0.001), CT (F = 72; p<0.001) and CF (F = 40; p<0.001). An interaction between food and dental status was shown for CC (F = 72; p<0,001); CT (F = 74; p<0,001) but not for CF (F = 0.74, p = 0.65). Both groups with impaired dental status (PD and DW groups) had higher mean CC and CT values than the FD group (SNK p<0.05). For both sieved foods (carrot and peanuts), there were significant intergroup differences among mean pre-surgical D50 values according to dental status (F = 33, p<0.001) and types of food (F = 62, p<0.001); PD and DW groups showed higher D50 values than the FD group (SNK, p<0.05).

After surgery, chewing time and the number of chewing cycles for all foods increased for all groups but not statistically significant for jelly. Student's paired *t*-test comparisons showed that the differences between mean pre- and post-surgical CT and CC values were not significant for jelly, nor for banana and apple in the DW group. Mean CF values did not change after surgery, whatever the dental status or food, excepted for peanuts in PD group. Despite increased chewing activity for carrot in all groups, the resulting changes in bolus granulometry depended on dental status. Mean D50 values remained as fine and as coarse as they were before surgery in groups FD and DW respectively. For group PD, mean D50 values decreased significantly after surgery, reaching values close to the bolus granulometry for fully dentate patients. Mean D50 values for peanuts remained unchanged after surgery, in both PD and DW groups and increased significantly in group FD (p<0.01).

## Discussion

This is the first study to assess variation in kinematic and granulometric parameters of mastication in obese people following bariatric surgery. It demonstrated that after gastric bypass surgery, the ability of obese patients to increase chewing time and number of chewing cycles depends on both dental status and type of food. Moreover, it showed that the effects of this adaptive chewing behaviour on bolus granulometry are also food and dental status-dependent. As expected in hypothesis 1, fully dentate patients increased their chewing activity and this change has no effect on carrot bolus granulometry. This result is representative of a healthy chewing pattern [29]. When optimal bolus granulometry and substantial cohesiveness and plasticity are reached, increasing the number of chewing cycles and time has no effect. The impact of changing mastication pattern is very different in patients who are dentally impaired. Pre-operative bolus granulometry was just as high in obese patients who had a mild FU deficit (PD group) as in those who wore a denture (DW group). Mean carrot bolus granulometry is close to the cut-off value of 4000 µm characterizing the upper limit of normal median particle size for carrots in a population of adults with good oral health [25]. The chewing function was deficient in both groups before surgery. Despite this deficiency, both PD and WD groups of patients received surgery and were asked to increase chewing time and the number of chewing cycles. After surgery, both groups complied with the advice, except for jelly. Hypothesis 2 was also confirmed, as partially dentate patients increased their chewing activity and the granulometry of their carrot bolus was considerably reduced, nearing the level observed in the bolus of fully dentate patients. Finally, denture wearers failed to change their chewing activity for banana, jelly and apple while they significantly increased their chewing activity for carrot and peanuts. This motion had no effect on the bolus granulometry of carrot, which remained insufficiently reduced compared with the carrot bolus produced by the fully dentate patients.

### BMI variations and dental status

This study was conducted under experimental conditions that did not allow the evaluation of spontaneous changes in either food intakes, satiety feelings or nutrient availability. The BMI variations recorded three months after surgery ranged were in the same range as those reported in other studies [37], [38] and did not differ between groups with different dental status, despite a difference in chewing behaviour and bolus granulometry. To explain the lack of difference in chewing behaviour with different BMI values, it could be argued that most of the variation in the post-surgery period is directly related to surgery. The consequences of dental status on weight and nutrition in obese patients after BS need to be evaluated over longer periods than 3 months after surgery. The modest sample size of the cohort and the low follow-up rate are the limitations for such analyses, although BMI variation was not the primary goal of the study.

### Effects of food properties on ability to change chewing

Hypothesis 2 was verified for carrot bolus but not for peanuts. After BS, the fully dentate subjects produced a peanut bolus with a higher median particle size despite having chewed peanuts for a longer time and for a higher number of cycles than before BS. The influence of food characteristics on the chewing process has already been described [28], [29], [39]. There are other food bolus characteristics than particle size that may help explain the time chosen for swallowing. The moderate correlation between the number of cycles and pre-swallow d50 that was seen in several studies [40], [41] may also reflect a need to reach certain rheological states, for example of viscosity, cohesiveness or stickiness, independently of particle size, in the final bolus [42], [43]. During chewing, the food pieces are reduced in size while saliva is produced to moisten the food until it reaches the ideal plasticity and cohesiveness for swallowing. Dry and hard products require more chewing cycles before swallowing than moist and soft products [24]. Peanut contain less water than carrot (around 0.5% vs 88.3 % respectively), and the consequences on bolus composition of an increase in chewing activity differ between carrot and peanuts. The increase in carrot chewing could produce juice that compensates for the additional decrease in particle size in order to satisfy the required plasticity of the pre-swallowing carrot bolus. This phenomenon did not occur with peanuts. In the absence of liquid release from the food, an increase in peanut comminution would lead to an increase of the viscosity of the peanut bolus, incompatible with the swallowing threshold. Faced with this problem, it could be hypothesized that the subjects adopt another strategy, consisting of replacing the “comminution-cycles” (chewing cycles that aim to crush the food between the teeth and to reduce the particle size) with “manipulation-cycles” (chewing cycles that aim to manipulate the food between the tongue and the palate, and to mix it with saliva and food juices). During manipulation-cycles, the peanuts particles are not crushed while saliva production is stimulated to allow the bolus to reach the ideal rheological properties to be swallowed. Each of these different strategies could possibly be interpreted as chewing cycles on video recordings. If this hypothesis could be verified for fully dentate subjects in further studies, the inability of both PD and DW groups to adapt their chewing behaviour to the food properties suggests again that dentally impaired patients with obesity should restrict their range food choices.

### Ability for changing kinematics

The ability to increase chewing activity following surgery results from a voluntary, conscious motion that depends on both dental status and food. Both groups with natural teeth (PD and FD groups) increased chewing time and the number of chewing cycles for carrot, peanut, apple and banana while they did not change significantly for jelly, as was also the case for denture wearers for apple and banana. Edentulous individuals lack an important source of tactile sensory input to the central nervous system (CNS), the periodontal mechanoreceptors. Periodontal mechanoreception provides feedback on the magnitude, direction, and rate of occlusal load application for sensory perception and motor function. In addition, intradental mechanoreception provides subtle modulation of occlusal loading for further refinement of neuromotor control of jaw function. When teeth are lost, these fine proprioceptive control mechanisms are absent. The presence or absence of periodontal mechanoreception necessarily has a direct bearing on tactile discrimination. [44]. It has been shown that the discrimination threshold for micro-thickness detection is lower for natural teeth compared with prosthetic teeth. This threshold is clearly dependent on the precision provided by periodontal mechanoreception (8–20 µm), which is higher for osseointegrated implant bridges (50 µm) and significantly higher for complete-denture wearers (100 µm). It therefore appears that denture wearers cannot modify their chewing strategy with soft samples because their ability to discriminate is poor, particularly with brittle foods such as carrots and peanuts. Furthermore, wearing a denture could modify the lingual praxis required during the ingestion of soft foods such as apple and banana, particularly for upper dentures with a resin palate.

### Improving dental status or changing food texture

Interactions of both food texture and dental status on chewing abilities have implications for the preventive /or therapeutical choices that could concern obese patients facing BS. Including a patient with fewer than six natural FUs in a therapeutic nutritional programme could expose him or her to a high risk of failure to chew, with adverse consequences on dietary behaviour. The solutions for compensating for missing teeth and restoring chewing function imply economic resources (for example, for implants and fixed prosthetics), that are not available for the majority of obese patients [45], [46]. The alternative to efficient mastication is artificial food comminution. Under these oral and economic conditions, the food texture would have to be adapted to the dental status. In some borderline cases, a rational approach would be to propose mixed or soft foods. These solutions could in turn induce modifications in eating behaviour that would have to be monitored and managed. Advice for preparing meals with mixed food may not be pertinent as it has low sensory, particularly textural, properties. The cephalic phase of digestion could therefore be negatively affected. Further research is needed to develop and improve such foods that should be, at the same time be sufficiently plastic and cohesive to be swallowed and sufficiently hard and resistant to induce sensorial inputs and muscular activity in order to trigger the cephalic reflex for initiating digestion.

### Increasing chewing or food manipulation for improving nutrition

Considering that increased chewing activity has no effect on bolus granulometry in either fully dentate patients or denture wearers, giving instructions to increase chewing seems to lack a rationale for such patients. The cephalic phase of digestion refers to a set of physiological, endocrine and autonomous responses of the digestive system which result from the stimulation of the sensory systems in the oropharyngeal cavity. Since Pavlov's work showing that sensory exposure to food in dogs triggers the secretion of gastric acid and pepsin, it has since become common knowledge that chemo-sensory stimuli exert a great influence on metabolism during food intake. The dorsal medulla oblongata contains the hub of the central nervous system that produces vagal cephalic-phase reflexes. The preganglionic motor neurons controlling these cephalic responses of digestion and metabolism are organized in longitudinal columnar subnuclei in the dorsal motor nucleus of the vagus nerve innervating the gastrointestinal tract [47].These mechanisms can be activated either directly by visceral afferents [47]–[49] or indirectly by peripheral inputs from the oral receptors that activate the brain centres. During manipulation of food in the mouth, the sensory properties perceived by receptors localized in the oral mucosa and around the teeth are conveyed by the trigeminal (Vth) nerve, while somatic sensory information from the pharynx and the posterior part of the tongue and taste information is transmitted by the glossopharyngeal (IXth) and vagus (Xth) cranial nerves.

Cephalic phase reflexes are designed to optimize and amplify gastrointestinal responses. The application of therapeutic mastication has proven effective in reducing visceral fat in leptin-deficient and leptin-resistant obese animals [50]. In humans, it has been shown that increasing the number of chewing cycles during almond mastication could increase the bioaccessibility of lipids from the nut, thereby increasing the amount of energy available to the body, thus contributing to a positive energy balance. In contrast, the increased presence of lipids in the small intestine may result in increased secretion of several hormones associated with stronger feelings of satiety [51]. In elderly subjects, it was demonstrated that post-prandial whole-body protein metabolism after a meat meal is influenced by chewing efficiency [52] and that oral functional training might activate the feeding function, possibly facilitating nutrient bioavailability [53]. Certain cephalic phase reflexes are also expected to fulfil a role in feeding behaviour by acting on the mechanisms of hunger and satiety [54], [55]. Eating with a pause between bites significantly reduces energy intake compared with eating with a soupspoon and taking pauses between spoonfuls and without a pause [56]. It was shown in an experimental setting that eating with small bite sizes rather than with large bite sizes and increasing oral processing time significantly decreases food intake [57].

Increasing the number of chewing cycles leads to an increase in the time food is manipulated in the mouth that, in turn, could possibly trigger the cephalic reflexes. Consequently, advice to increase chewing activity is pertinent for obese patients with healthy teeth, although this motion would have no effect on the bolus granulometry. For obese patients with poor dental status, the advice on increasing chewing should be changed for advices to firstly, recommendations about preparing soft foods and, secondly, guidance on increasing the manipulation time of food in the mouth. An increase in time for soft food manipulation in the mouth could be part of the optimisation of the nutrition process. This last solution should be explored in further clinical studies.

### Conclusion

Patients with obesity complied with the advice on increasing chewing after bariatric surgery, whatever their dental status. The interactions between dental status and food properties may affect the consequences of changes on chewing activities on the pre-swallowed bolus composition. The relationships between dental status, chewing behaviour, feeding attitudes and nutrition are complex and further studies are required to obtain a better understanding of the impact of dental status on feeding behaviour and nutrition in obese patients. In further studies, collection of data including not only granularity but also rheological properties of the food bolus should be undertaken to gain a better understanding of the link between physiological properties and final D50 values observed at the swallowing time. Despite these limitations, this study emphasizes the need for a comprehensive approach regarding patients scheduled for gastric bypass surgery, including the use of dental status as a predictive criterion for failure or success in changing chewing behaviour and feeding attitudes. The debate is now open on supplementing guidelines for therapeutic decisions that could be applied to obese patients with poor dental status.

## References

[1] National Institute of Health (1991). http://consensus.nih.gov/1991/1991GISurgeryObesity084html.htm.

[2] Santry HP, Gillen DL, Lauderdale DS (2005). Trends in bariatric surgical procedures..

[3] Buchwald H, Oien DM (2009). Metabolic/bariatric surgery Worldwide 2008.. Obes Surg.

[4] Dixon JB (2009). Obesity and diabetes: the impact of bariatric surgery on type-2 diabetes.. World J Surg.

[5] Buddeberg-Fischer B, Klaghofer R, Krug L, Buddeberg C (2006). Physical and psychosocial outcome in morbidly obese patients with and without bariatric surgery: a 4 1/2-year follow-up.. Obes Surg.

[6] Agence Nationale d′Accréditation et d′Evaluation enSanté (2001). http://www.has-sante.fr/portail/upload/docs/application/pdf/chirurgie_obesite_rap.pdf.

[7] N′gom PI, Woda A (2002). Influence of impaired mastication on nutrition.. J Prosthet Dent.

[8] Pera P, Bucca C, Borro R, Bernocco C, De Lillo  (2002). Influence of mastication on gastric emptying.. J Dent Res.

[9] Lipsitz LA, Ryan SM, Parker A, Freeman R, Wei  JY (1993). Hemodynamic and autonomic nervous system responses to mixed meal ingestion in healthy young and old subjects and dysautonomic patients with postprandial hypotension.. Circulation.

[10] Kaneko H, Sakakibara M, Mitsuma T, Morise K (1995). Possibility of postprandial electrogastrography for evaluating vagal/nonvagal cholinergic activity in humans, through simultaneous analysis of postprandial heart rate variability and serum immunoreactive hormone levels.. Am J Gastroenterol.

[11] Momose T, Nishikawa J, Watanabe T, Sasaki Y, Senda M (1997). Effect of mastication on regional cerebral blood flow in humans examined by positron-emission tomography with o-labelled water and magnetic resonance imaging.. Archs Oral Biol.

[12] Farella M, Bakke M, Michelotti A, Marotta G, Martina R (1999). Cardiovascular responses in humans to experimental chewing of gums of different consistencies.. Arch Oral Biol.

[13] De Araujo IE, Rolls ET (2004). Representation in the human brain of food texture and oral fat.. J Neurosci.

[14] Hasegawa Y, Sakagami J, Ono T, Hori K, Zhang M et al (2009). Circulatory response and autonomic nervous system activity during gum chewing.. Eur J Oral Sci.

[15] Mattes RD (1997). Physiological responses to sensory stimulation by food: nutritional implications.. J Am Diet Assoc.

[16] Power ML, Schulkin J (2008). Anticipatory physiological regulation in feeding biology: cephalic phase responses.. Appetite.

[17] Bellisle F, Le Magnen J (1981). The structure of meals in humans : eating and drinking patterns in lean and obese subjects.. Physiol Behav.

[18] Bellisle F, Rolland-Cachera MF, Deheeger M, Guilloud-Bataille M (1988). Obesity and food intake in children : evidence for a role of metabolic and/or behavioral daily rythms.. Appetite.

[19] De Jonge L, Bray GA (1997). The thermic effect of food and obesity: a critical review.. Obes Res.

[20] De Jonge L, Garrel DR (1997). Role of the autonomic nervous system in the thermogenic response to food in lean individuals.. Am J Physiol.

[21] Hashkes PJ, Gartside PS, Blondheim SH (1997). Effect of food palatability on early (cephalic) phase of diet-induced thermogenesis in noobese and obese man.. Int J Obes Relat Metab Disord.

[22] Veyrune JL, Miller CC, Czernichow S, Ciangura CA, Nicolas E (2008). Impact of morbid obesity on chewing ability.. Obes.

[23] Prinz JF, Lucas PW (1997). An optimization model for mastication and swallowing in mammals.. Proc Biol Sci.

[24] van der Bilt A Assessment of mastication with implications for oral rehabilitation:areview.. J Oral Rehabil.

[25] Woda A, Nicolas E, Mishellany-Dutour A, Hennequin M, Mazille MN (2010). The masticatory normative indicator.. J Dent Res.

[26] Mishellany A, Woda A, Labas R, Peyron MA (2006). The challenge of mastication: preparing a bolus suitable for deglutition.. Dysphagia.

[27] Woda A, Mishellany A, Peyron MA (2006a). The regulation of masticatory function and food bolus formation.. J Oral Rehabil.

[28] Agrawal KR, Lucas PW, Bruce IC, Prinz JF (1998). Food properties that influence neuromuscular activity during human mastication.. J Dent Res.

[29] Woda A, Foster K, Mishellany A, Peyron MA (2006b). Adaptation of healthy mastication pertaining to the individual or to the food.. Physiol Behav.

[30] Mishellany-Dutour A, Renaud J, Peyron MA, Rimek F, Woda A (2008). Is the goal of mastication reached in young dentates, aged dentates, and aged denture wearers?. Brit J Nut.

[31] Slagter AP, Bosman F, van der Glas HW, van der Bilt A (1993). Human jaw-elevator muscle activity and food comminution in the dentate and edentulous state.. Arch Oral Biol.

[32] Peyron MA, Lassauzay C, Woda A (2002). Effects of increased hardness on jaw movement and muscle activity during chewing of visco-elastic model foods.. Exp Brain Res.

[33] Peyron MA, Blanc O, Lund JP, Woda A (2004). Influence of age on adaptability of human mastication.. J Neurophysiol.

[34] World Health Organisation (1995). The use and interpretation of anthopometry..

[35] Hennequin M, Allison PJ, Veyrune JL, Faye M, Peyron MA (2005). Clinical evaluation of mastication: validation of video versus electromyography.. Clin Nutr.

[36] Nicolas E, Veyrune JL, Lassauzay C, Peyron MA, Hennequin M (2007). Validation of video versus electromyography for chewing evaluation of the elderly wearing a complete denture.. J Oral Rehabil.

[37] Buchwald H, Avidor Y, Brauwald E, Jensen MD, Pories W (2004). Bariatric surgery, a systematic review and meta-analysis.. JAMA.

[38] Maggard MA, Shugarman LR, Suttorp M, Maglione M, Sugerman HJ (2005). Meta-analysis: surgical treatment of obesity.. Ann Intern Med.

[39] Yven C, Guessama S, Chaunier L, Della Valle G, Salles C (2010). The role of mechanical properties of brittle airy foods on the masticatory performance.. J Food Eng.

[40] Peyron MA, Mishellany A, Woda A (2004). Particle size distribution of food boluses after mastication of six natural foods.. J Dent Res.

[41] Fontijn-Tekamp  FA,  Van der Bilt A, Abbink  JH, Bosman F (2004). Swallowing threshold and masticatory performance in dentate adults.. Physiology and Behavior.

[42] Prinz  JF, Lucas  PW (1995). Swallow thresholds in human mastication.. Archives of Oral Biology.

[43] Seo  HS, Hwang IK,  Han TR,  Kim IS ( 2007). Sensory and instrumental analysis for slipperiness and compliance of food during swallowing.. Journal of Food Science.

[44] Klineberg I, Murray G (1999). Osseoperception: sensory function and proprioception.. Adv Dent Res.

[45] Vernay M, Malon A, Oleko A, Salanave B, Roudier C et al (2009). Association of socioeconomic status with overall overweight and central obesity in men and women: the French Nutrition and Health Survey 2006.. BMC Public Health.

[46] De Saint Pol T (2009). Evolution of obesity by social status in France, 1981–2003.. Econ Hum Biol.

[47] Powley TL (2000). Vagal circuitry mediating cephalic-phase responses to food.. Appetite.

[48] Brand JG, Cagan RH, Naim M (1982). Chemical senses in the release of gastric and pancreatic secretions.. Ann Rev Nutr.

[49] Giduck SA, Threatte RM, Kare MR (1987). Cephalic reflexes: their role in digestion and possible roles in absorption and metabolism.. J Nutr.

[50] Sakata T, Yoshimatsu H, Masaki T, Tsuda K (2003). Anti-obesity actions of mastication driven by histamine neurons in rats.. Exp Biol Med.

[51] Cassady BA, Hollis JH, Fulford AD, Considine RV, Mattes RD (2009). Mastication of almonds: effects of lipid bioaccessibility, appetite, and hormone response..

[52] Rémond D, Machebeuf M, Yven C, Buffière C, Mioche L et al (2007). Postprandial whole-body protein metabolism after a meat meal is influenced by chewing efficiency in elderly subjects.. Am J Clin Nutr.

[53] Kikutani T, Enomoto R, Tamura F, Oyaizu K, Suzuki  A (2006). Effects of oral functional training for nutritional improvement in Japanese older people requiring long-term care.. Gerodontology.

[54] LeBlanc J, Soucy J (1996). Interactions between postprandial thermogenesis, sensory stimulation of feeding, and hunger.. Am J Physiol.

[55] Harthoorn LF, Dransfield E (2008). Periprandial changes of the sympathetic-parasympathetic balance related to perceived satiety in humans.. Eur J Appl Physiol.

[56] Andrade AM, Greene GW, Melanson KJ (2008). Eating slowly led to decreases in energy intake within meals in healthy women.. J Am Diet Assoc.

[57] Zijlstra N, de Wijk RA, Mars M, Stafleu A, de Graaf C (2009). Effect of bite size and oral processing time of a semisolid food on satiation.. Am J Clin Nutr.

